# Design, Synthesis, and Molecular Docking Studies of Curcumin Hybrid Conjugates as Potential Therapeutics for Breast Cancer

**DOI:** 10.3390/ph15040451

**Published:** 2022-04-06

**Authors:** Siva S. Panda, Queen L. Tran, Pragya Rajpurohit, Girinath G. Pillai, Sean J. Thomas, Allison E. Bridges, Jason E. Capito, Muthusamy Thangaraju, Bal L. Lokeshwar

**Affiliations:** 1 Department of Chemistry and Physics, Augusta University, Augusta, GA 30912, USA; queentran29@gmail.com (Q.L.T.); seanjosephthomas@gmail.com (S.J.T.); jcaps79@gmail.com (J.E.C.); 2 Department of Biochemistry and Molecular Biology, Augusta University, Augusta, GA 30912, USA; pragyaraj2018@gmail.com (P.R.); allison.bridges@ngu.edu (A.E.B.); blokeshwar@augusta.edu (B.L.L.); 3 Georgia Cancer Center, Augusta University, Augusta, GA 30912, USA; 4 Discovery Chemistry, Nyro Research India, Kochi 682021, India; giribio@gmail.com; 5 Department of Medicine, Medical College of Georgia, Augusta University, Augusta, GA 30912, USA

**Keywords:** curcumin, DCA, amino acid, molecular hybridization, molecular docking, breast cancer

## Abstract

Curcumin (CUR) has received great attention over the past two decades due to its anticancer, anti-inflammatory, and antioxidant properties. Similarly, Dichloroacetate (DCA), an pyruvate dehydrogenase kinase 1 (PKD1) inhibitor, has gained huge attention as a potential anticancer drug. However, the clinical utility of these two agents is very limited because of the poor bioavailability and unsolicited side effects, respectively. We have synthesized fusion conjugates of CUR and DCA with an amino acids linker to overcome these limitations by utilizing the molecular hybridization approach. The molecular docking studies showed the potential targets of Curcumin-Modified Conjugates (CMCs) in breast cancer cells. We synthesized six hybrid conjugates named **CMC1-6**. These six CMC conjugates do not show any significant toxicity in a human normal immortalized mammary epithelial cell line (MCF10A) in vitro and C57BL/6 mice in vivo. However, treatment with **CMC1** and **CMC2** significantly reduced the growth and clonogenic survival by colony-formation assays in several human breast cancer cells (BC). Treatment by oral gavage of a transgenic mouse BC and metastatic BC tumor-bearing mice with **CMC2** significantly reduced tumor growth and metastasis. Overall, our study provides strong evidence that CUR and DCA conjugates have a significant anticancer properties at a sub-micromolar concentration and overcome the clinical limitation of using CUR and DCA as potential anticancer drugs.

## 1. Introduction

Breast cancer (BC) is the second most common cancer in women, with an estimated 290,560 cases in 2022 and 43,780 deaths. BC is a complex biological disease that becomes lethal as it progresses, with limited options for curing it beyond the early stage of localized cancer. Like many other human cancers, BC results from significant alterations in genetic and epigenetic mechanisms and targeting multiple signaling pathways in growth and malignant progression towards incurable lethal disease [1]. Targeting a single-cell signaling pathway is unlikely to prevent or cure BC. Combination therapy (adjuvant therapy) is a current strategy for BC treatment and prevention of its progression [2]. The chemotherapeutic drugs for treating BCs, only target rapidly growing tumor cells but are less lethal to cancer stem cells [3]. However, these anticancer drugs inevitably produce severe, systemic toxicities in patients after chemotherapy. The emergence of resistance to drugs used in chemotherapy and rapid regrowth of tumors is another limitation of chemotherapy. Therefore, the development of novel small molecules that can be used as a non-toxic adjuvant to chemotherapy is a promising strategy for prolonging the quality and longevity of BC patients.

**Figure 1:**
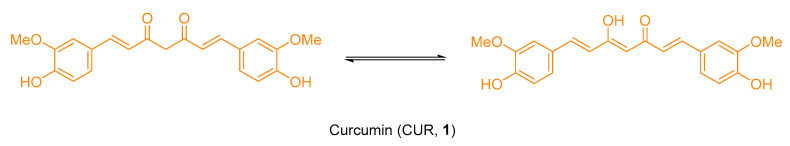
Keto-enol tautomeric forms of curcumin (CUR).

Curcumin (CUR, **1**) (Figure 1), the spice turmeric’s major ingredient, is well-documented for its anti-inflammatory and pro-apoptotic activities against many solid tumors [4,5]. However, CUR is ineffective as an anticancer agent because of its low bioavailability, even at high pharmacological doses [6]. Almost two decades of scientific work on CUR has minimal utility in treating human cancers. Several chemical approaches are utilized, such as optimizing the pharmacological formulations’ nano-formulation [7], prodrugs [8], and molecular modifications [9] to increase the bioactivity and potency of CUR. Biological evaluation of the modified form of CUR has mainly been limited to in vitro testing and seldom for their bioavailability by multiple routes, such as oral, intravenous, and intraperitoneal administration [10].

Dichloroacetate (DCA) is another leading natural product against BC since 2007 [11]. DCA targets the pyruvate-led glycolytic pathway in cancer cells because of its structural similarities with pyruvate. DCA could trigger apoptosis of human BC cells, and this is very effective and shows the synergistic effect when used in combination with other drugs [12,13,14,15]. A recent report suggests that DCA treatment led to a significant increase in ROS production (up to 15-fold) in hypoxic cancer cells but not in aerobic cells [16]. However, its use in the treatment of BC has been absent as some studies showed unusual adverse toxicity [17].

**Figure 2:**
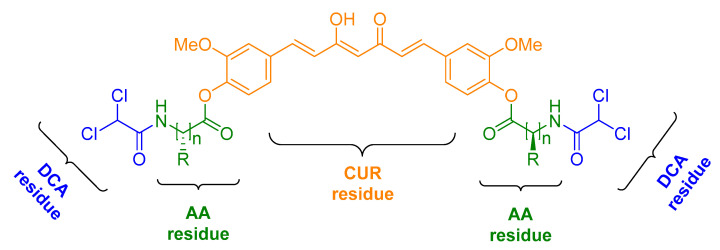
Target hybrid conjugates.

Several CUR analogs and conjugates have been reported upon in the last decade for their anticancer properties [18,19]. Most of the CUR analogs and conjugates are studied for their anticancer properties against various cell lines [18,19,20]. However, very few reports on animal studies on synthesized compounds administered orally at a very high dose [21] or other routes [22,23]. We adopted a molecular hybridization approach to synthesize our proposed molecules (Figure 2). Our synthesized compounds overcome the barriers associated with CUR and DCA.

Among various rational drug design strategies, molecular hybridization (conjugation of two or more anticancer molecules via a covalent bond) is an effective and efficient tool for developing new drug candidates for BC. Furthermore, the molecular hybrids could also overcome drug resistance, lower the risk of drug–drug interactions, have cost-effective, synergistic effects, improve interactions with multiple pharmacological sites, and minimize redundant side effects [24,25,26]. Previously, we reported various conjugates with amino acids (AAs) of enhanced lipophilic properties, which retain or increase the biological properties with respect to the parent molecule(s) [27]. To aid in the discovery of new drug developments, we have actively explored the molecular hybridization approach to synthesize hybrid conjugates using CUR, DCA, and AAs as building blocks.

In the present study, we have designed and synthesized a set of novel CUR–DCA conjugates with potential anticancer properties against BC using a conjugate chemistry approach employing amino acids as linkers. The well-characterized synthesized hybrid conjugates were screened against a human normal immortalized mammary epithelial cell line (MCF10A), human ER + BC cell line (T47D), and TNBC cell line (MB231) using MTT cell proliferation and colony formation assays. The most potent conjugate was further validated in the spontaneous mouse mammary tumor model (MMTV–PyMT–Tg).

## 2. Results and Discussion

For our present study, we tried several reaction conditions and coupling reagents to establish an optimized condition to prepare the desired set of CMC conjugates of CUR and DCA with an AA linker in pure form with chiral integrity. We successfully synthesized six CMC conjugates designed using the molecular hybridization approach (**CMC1–6**, Figure 1) and fully characterized by spectral studies.

To better understand the role of the amino acids as a linker, we have also prepared a conjugate of DCA and CUR (**CMC1**) without any linker. For the rest of the conjugates, we used glycine (**CMC2**). *L*-alanine (**CMC3**). β–alanine (**CMC4**). *L*-phenylalanine (**CMC5**) and γ–aminobutyric acid (**CMC6**). All purely synthesized conjugates were screened against BC cell lines, and the most potent ones (**CMC1** and **CMC2**) were further considered for animal studies. The toxicity study was also carried out against a human normal immortalized mammary epithelial cell line (MCF10A) and normal C57BL/6 mice. In addition, the molecular docking studies support the experimental observations.

### 2.1. Chemistry

**Scheme 1:**
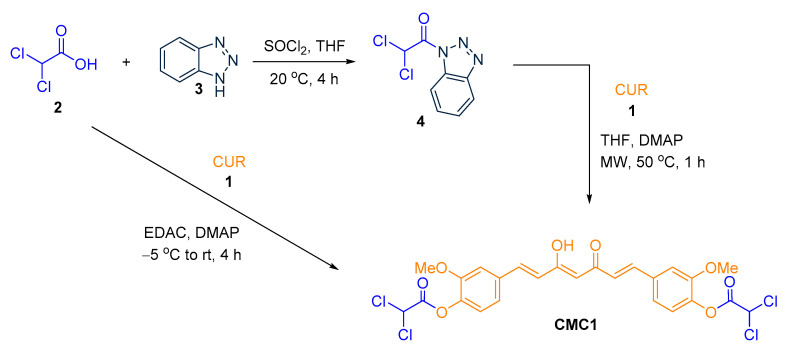
Synthesis of DCA–curcumin conjugate **CMC1**.

The current study is focused on evaluating some CUR–DCA conjugates with and without an amino acid linker to correlate activity, structure, and bioavailability. To synthesize the CUR–DCA conjugates without any linker, DCA was activated by benzotriazole **3** using our previously reported method [28]. The benzotriazole activated DCA **4** treated with CUR in the presence of 4-(dimethylamino)pyridine (DMAP) in tetrahydrofuran (THF) under microwave irradiation. We also synthesized **CMC1** by using an alternative route where DCA was treated with CUR in the presence of *N*-(3-Dimethylaminopropyl)-*N*′-ethyl carbodiimide hydrochloride (EDAC) and DMAP in DCM to obtain the hybrid conjugate of DCA and CUR after recrystallizing with ethanol (Scheme 1). The reaction condition was optimized in our previous report [29]. We found that the alternative route for preparing **CMC1** was more efficient in yield and purity.

**Scheme 2:**
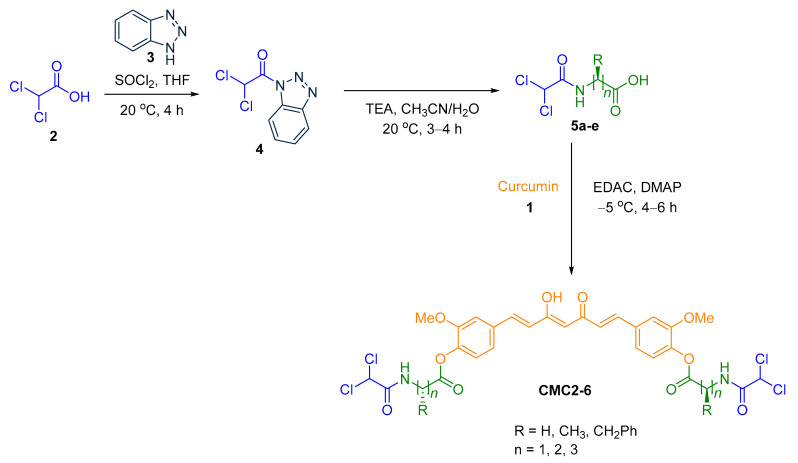
Synthesis of DCA–curcumin conjugate with amino acid as linker **CMC2–6**.

To introduce amino acid as a linker in the conjugate, the benzotriazole activated DCA **4** was treated with amino acids in the presence of triethylamine (TEA) in aqueous acetonitrile at room temperature to form the DCA–amino acid conjugates **5a–e** [30]. Conjugates **5a–e** is further coupled with CUR **1** under optimized reaction conditions to yield the hybrid conjugates **CMC2–6** (Scheme 2).

**Figure 3:**
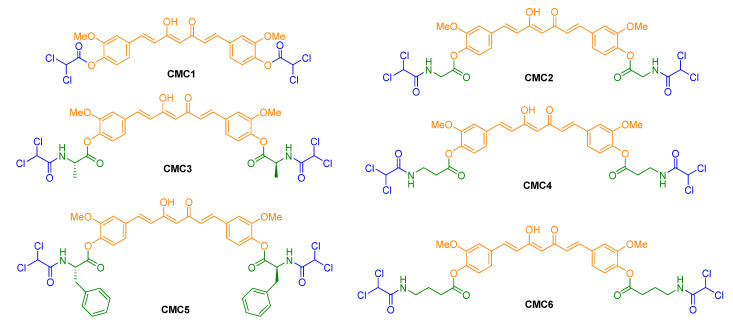
Structures of synthesized CUR and DCA hybrid conjugates **CMC1–6**.

We successfully synthesized the following six curcumin conjugates (Figure 3) in pure form, which were fully characterized by spectral studies.

### 2.2. Biology

**Figure 4:**
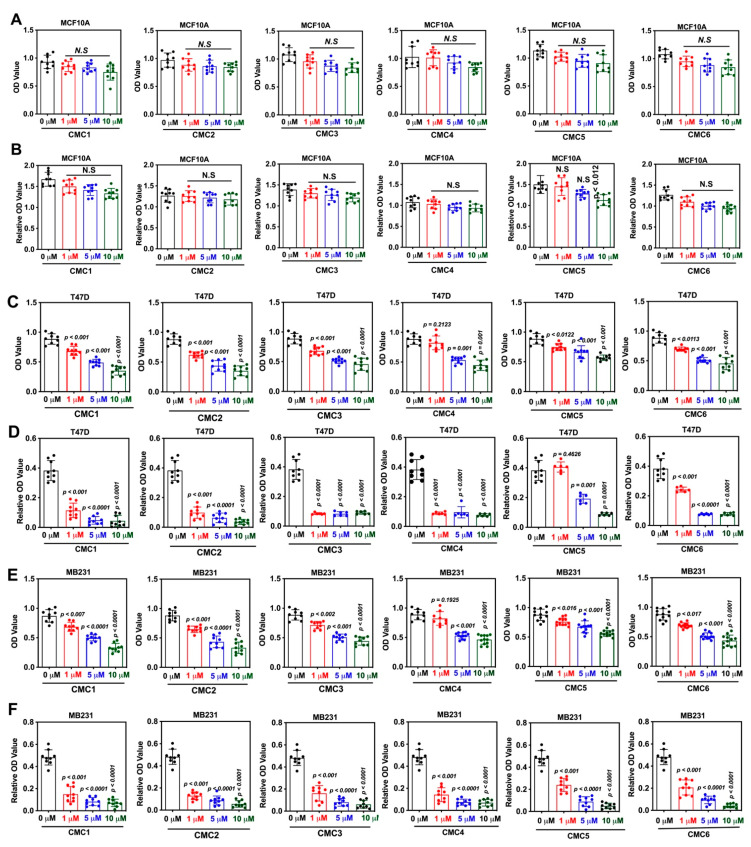
CMC conjugates inhibit human BC cell growth at submicromolar concentration. (**A**,**C**,**E**) MTT and (**B**,**D**,**F**) colony formation assays were carried out in MCF10A, T47D, and MB231 cells with **CMC1–6** at different concentrations (0, 1, 5, and 10 μM).

We tested the antitumor potential of all six CMC conjugates (**CMC1–6**) in a human normal immortalized mammary epithelial cell line (MCF10A), human ER + BC cell line (T47D), and TNBC cell line (MB231) using MTT (3-(4,5-dimethylthiazol-2yl)-2,5-diphenyltetrazolium bromide) cell proliferation and colony formation assays, as described in our previous manuscripts [30,31]. Our results show that none of these CMC conjugates inhibited cell proliferation in human normal immortalized cell lines, but most of these CMC conjugates are effectively inhibited cell proliferation in both ER + BC and TNBC cell lines at a nanomolar concentration (Figure 4). 

**Figure 5:**
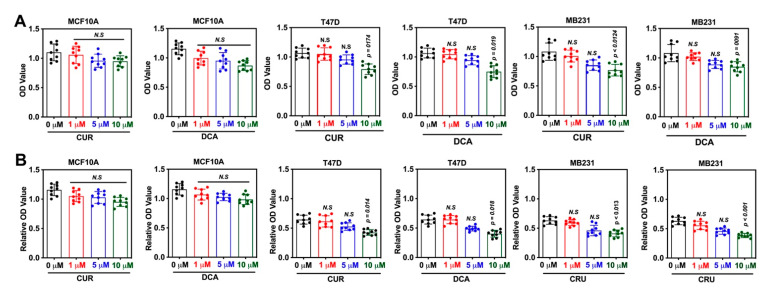
CUR and DCA inhibit cell viability and colony formation, to a lower extent, in human BC cells. (**A**) MTT, and (**B**) colony formation assays were carried out in MCF10A, T47D, and MB231 cells with CUR and DCA at different concentrations (0, 1, 5, and 10 μM).

We also tested the antitumor potential of the parent CUR and DCA compounds in these cell lines (MCF10A, T47D, and MB231). However, CUR and DCA inhibited cell viability and colony formation in T47D and MB231 cells (Figure 5) but not in MCF10A cells. Based on these results, we calculated EC_50_ values for these conjugates and found that most of these compounds inhibited BC cell growth at submicromolar concentrations (Table 1). These observations provide a strong rationale to test the hypothesis that CMC conjugates would have high antitumor potential and, therefore, it is imperative to establish the antitumor potential of these compounds in BC growth and metastasis. Several curcumin analogs were synthesized and screened against various cancer cell lines according to the literature data. The reported data are all in the range of micromolar to molar concentration [21,23]. However, our synthesized conjugates showed potency at submicromolar concentrations. As shown in Table 1, all CMCs had very low activity against the normal breast epithelial cells, MCF10. The EC_50_ values of CMCs against MCF10A were about 8–16 times that against TNBC cell lines. These results show high tumor specificity.

### 2.3. Toxicity Studies

**Figure 6:**
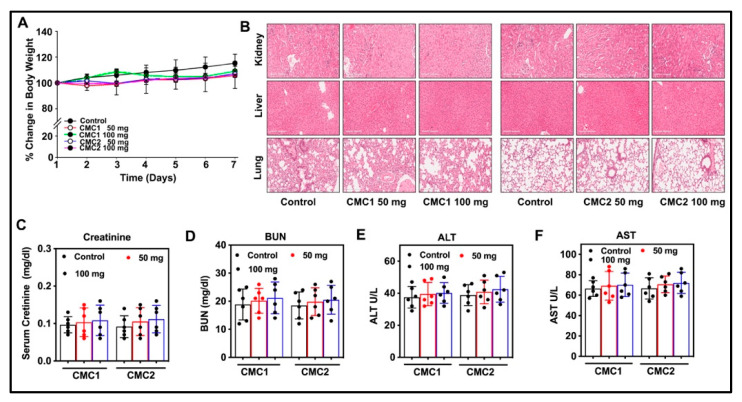
CMC conjugates are safer and do not show any contraindication. Normal C57BL/6 mice (*n* = 6) were treated with **CMC1** and **CMC2** at two different concentrations (50 and 100 mg/kg body) by oral gavage for 7 days. Bodyweight, (**A**) movement, hair loss, food, and water intake were monitored. At the end of the experimental period (7 days after the treatment), mice were euthanized, tissues (kidney, liver, and lung) were collected to monitor morphological changes by H&E staining. Representative images are shown at 20x magnification (**B**). Blood samples were collected to measure Creatinine (**C**), BUN (**D**), ALT (**E**), and AST (**F**) levels. Values are shown as the mean ± SD of 6 animals in each group.

We tested whether the synthesized conjugates are free of toxicity and safer to use in human normal cells and animal models. We treated human normal immortalized cell line (MCF10A) and normal C57BL/6 mice at different concentrations and time points. We found that none of these conjugates (**CMC1-6**) inhibited cell viability, measured by MTT assay, in human normal immortalized cell lines (Figure 4 and Figure 5). Similarly, treatment of normal C57BL/6 mice with two different concentrations (50 and 100 mg/kg body for 7 days) of **CMC1** and **CMC2** conjugates showed no changes in the body weight, morphology, kidney functions (serum creatinine and blood urea and nitrogen), and liver functions (ALT and AST) (Figure 6A–F). These observations clearly show that CMC conjugates are safer and free of toxicity.

### 2.4. Animal Studies

**Figure 7:**
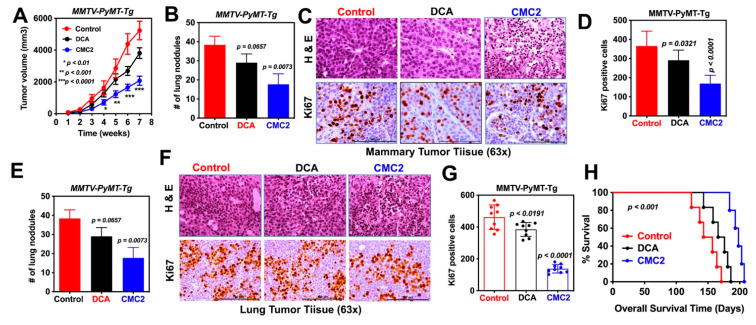
CMC2 treatment inhibits tumor growth in spontaneous mouse mammary tumor models. Six-week-old *MMTV–PyMT–Tg* mice were treated with DCA and **CMC2** (10 mg/kg body for 3× a week for 7 weeks. Control mice received PBS. (**A**) Tumor size was measured twice a week and tumor volume was calculated as described in our previous manuscript [31]. After seven weeks, mice were euthanized, tumor tissue was collected, and measured tumor weight (**B**). Tumor tissue sections were prepared and stained with H&E, and Ki67- and Ki67-positive cells were quantitated manually (**C**,**D**). Lung tissues were collected, visible lung nodules were manually counted (**E**). Lung tissue sections were prepared and stained with H&E and Ki67. The density of Ki67-positive cells per objective field was manually counted (**F**,**G**). Finally, we monitored the overall survival (**H**). Values are shown as mean ± SD of six mice in each group.

We chose and studied the antitumor potential of the most active CMC conjugates, **CMC2**, in a spontaneous mouse mammary tumor model (*MMTV–PyMT–Tg* mice). We chose *MMTV–PyMT–Tg* mouse mainly because the tumor formation and progression in this mouse is characterized by four different stages (hyperplasia, adenoma/mammary intra-epithelial neoplasia, early and late carcinoma) and also mimics human BC; the tumor develops first as ER-positive (ER^+^) but ultimately becomes ER-negative BC (ER^−^ BC) [31,32]. We randomly assigned 6-week-old *MMTV–PyMT–Tg* mice into two groups (6 mice in each); one control and one **CMC2** treated (10 mg/kg body, three times a week by oral gavage for 7 weeks). We measured the tumor volume twice a week. Tumor volume was calculated using the formula V = L × W^2^/2, where L represents the largest tumor diameter, and W represents the smallest tumor diameter. Mice were euthanized after seven weeks of treatment, and tumor tissues were collected. The total tumor weight was measured. Tumor tissue sections were prepared and stained with Hematoxylin and Eosin (H&E) for morphometric analysis and Ki67 for cell proliferation analysis. As shown in Figure 6, **CMC2** treatment significantly reduced tumor growth without affecting the normal body weight and organ histology (Figure 7A,B) and tumor weight (Figure 7B). **CMC2** treatment significantly reduced tumor growth by inhibiting tumor cell proliferation in the mammary tumor tissue (Figure 7C,D) and lung tumor tissue (Figure 7F,G), reducing lung nodules (Figure 7E) with increased overall survival rate (Figure 7H).

Overall, our studies provided evidence for the following three important observations: (**1**) CMC conjugates do not show any adverse side effects like kidney, liver, or lung toxicity. Therefore, these compounds are safe to use for clinical trials; (**2**) CMC conjugates show a potential antitumor activity in both luminal ER-positive (T47D cells) and basal triple-negative breast cancer (TNBC) cells by inhibiting cell growth and colony formation. (**3**) One of the CMC conjugates (**CMC2**) has a strong antitumor potential in vivo by inhibiting tumor growth in the GEM mouse model of BC (*MMTV–PyMT–Tg*), which mimics human BC [33,34].

### 2.5. Computational Studies

The experimental anticancer data was interesting and promising, which encouraged us to validate the experimental data by docking studies. According to a recent report, CUR inhibits 26S proteasome activity by directly inhibiting dual-specificity tyrosine-regulated kinase 2 (DYRK2) [35,36], and we deployed this target protein for our docking studies. The docking results interpret the most active conjugate of the six synthesized compounds to have a better docking score. Obtaining balanced pharmacokinetic (ADME—Absorption, Distribution, Metabolism, and Excretion) properties of drug-like molecules is one of the most difficult and challenging parts of the drug development process [37].

The execution of the molecular docking study is to identify whether CMC compounds modulate T47D and identify potential binding sites for a well-established ER- Breast cancer target (PDB ID:5ZTN). Prediction of binding sites was performed by a combinatorial analysis. Binding site prediction was made by conducting literature reviews on the DYRK2 target. Computational tools such as DoGSiteScorer and ScanProsite were used to predict the binding sites for the same. DoGSiteScorer reported a drug score of 81% having 41% non-polar, 28% polar, 18% of −ve, and 13% of +ve amino acids and including 225 interaction points within the binding pocket. Validation of binding sites was carried out by establishing a comparative analysis of binding sites obtained from all three sources. Predicted binding sites for DYRK2 include Ile, Ala, Lys, Phe, Leu, and Asp involved in the key binding interactions (Table 2).

Molecular docking studies were carried out by FlexX4, which exploits incremental construction algorithms to predict dock scores. The significance of the docking score implies how comfortable the ligand is interacting with the protein. Prediction of binding affinity and ligand efficiency (L.E) were performed by the HYDE algorithm [38]. Chain A of protein was considered for docking study since the amino acid residues present in the binding site were associated with chain A. The top 100 poses of the solutions were generated by considering three different stereo modes of ligands such as E/Z, R/S, and pseudo-R/S. The binding of ligand to protein is driven by the enthalpy-entropy-based hybrid approach.

Even though CUR has a good docking score and comfortably binds to the pocket of the protein target, the compound is not stable while considering desolvation terms and torsional alerts. On the other hand, **CMC2** has acceptable docking scores along with free binding affinity in agreement with desolvation terms and torsional alerts. Docking analysis revealed the selectivity of interactions with key amino acids, surface characteristics, including the regulatory mechanism of the DYRK2. To better characterize and make decisions on drug-like derivatives, we carried out pharmacokinetic studies to predict a few ADME properties to understand the liability. **CMC2** showed optimally balanced properties of aqueous solubility (Sol), HERG liability (HERG II), developmental toxicity (Dev. Tox.), P-glycoprotein substrate/non-substrate (P-gp), and 2D6 isoform of P450 affinity data. The violation of drug-likeness, the Lipinski rule, including oral bioavailability, could be overcome by lead optimization methods to design derivatives within the applicability domain of potency and all pharmacokinetic properties. The predicted ADME data looks promising (Table 3). Even though the orally administered animal studies provided preliminary results, we will investigate the blood serum of the treated animal at different intervals of time to analyze the presence of our conjugate and or the hydrolyzed products and communicate in the future.

In vitro studies confirmed the significant role of **CMC2** in eliciting anticancer activity. In silico studies conducted on synthesized hybrid conjugates reported the binding affinity, significant interactions as well as bioavailability of these novel compounds concerning curcumin. Out of six hybrid conjugates, **CMC2** exhibited a higher dock score, binding energy as well as ligand efficiency. The binding energy of curcumin was found to be −24 kJ/mol, ligand efficiency 0.22, and dock score of −29.24. However, **CMC2** exhibited a much higher range of these parameters, which indicates the likeliness of this compound to inhibit DYRK2. Even though the docking score of **CMC6** is considerably low, binding energy and ligand efficiency are comparable to **CMC2**. All conjugates showed significant interactions with DYRK2. The comparative analysis of binding interactions revealed the presence of H-bonds with two significant amino-acid residues, Leu231 and Asp295, in all the derivatives. The NH- group of Leu231 made H-bond interactions with the protein, while polar amino acid Asp295 contributes to making stronger interactions with the target protein by donating hydrogen atoms.

Bioavailability studies emphasize the significance of human intestinal absorption, affinity towards P450 isoform CYP2D6, developmental toxicity, hERG inhibition, and lipophilicity. Affinity toward the P450 isoform confirms the metabolic stability of compounds. A low/medium range of affinity is acceptable since higher affinity towards cytochrome P450 results in the decreased therapeutic value of lead-like compounds. This is due to the higher rate of conversion of compounds into metabolic end products before eliciting its therapeutic activity [39]. Developmental toxicity is highly undesirable since this could affect the entire homeostasis process. hERG is a gene encoding the alpha subunit of the potassium ion channel. Drug-induced inhibition of hERG results in the development of cardiac-related disorders [40]. Lipophilicity is an essential parameter depicting the permeability of lead-like molecules into biological membranes.

The curcumin reported for anticancer activity was found to inhibit hERG and possess developmental toxicity, which is not appreciable. However, the hybrid conjugate **CMC2** has the optimal balance for all the above-mentioned parameters. Hence, the potency of **CMC2** in executing anticancer activity is confirmed by in vitro and in silico approaches. **CMC3** got good bioavailability scores which are comparable to **CMC2**. All conjugates exhibited good intestinal absorption profiles, metabolic profiles, and lipophilicity. However, **CMC4**, **CMC5**, and **CMC6** were found to exhibit developmental toxicity, and **CMC5** was reported for hERG inhibition. Hence, future studies focusing on the optimization of these derivatives could bring the most promising lead molecules having anticancer activity.

## 3. Materials and Methods

Melting points were determined on a capillary tube melting point apparatus equipped with a digital thermometer. NMR spectra were recorded in DMSO-d6 on a Bruker NMR spectrometer operating at 500 MHz for 1H (with TMS as an internal standard) and 125 MHz for 13C. were performed on reverse phase gradient using Agilent (Santa Clara, CA, USA) 1200 series binary pump (G1312B), waters XTerra MS C18 (3.5 mm; 2.1–150 mm) þ Phenomenex C18 security guard column (2–4 mm) using 0.2% acetic acid in H2O/methanol as mobile phases; wavelength ¼ 254 nm; and mass spectrometry was done with 6220 Agilent (Santa Clara, CA, USA) TOF in electrospray ionization (ESI) mode with a positive and negative method in both Profile and Centroid mode. HPLC studies were done with 6120 Agilent (quadrupole LC/MS) [29].

### 3.1. Synthesis of CUR–DCA Hybrid Conjugate (**CMC1**)

A dried round bottom flask containing a small stir bar was charged with CUR (1.0 equivalent) and DCA (2.0 equivalent) dissolved in DCM (5 mL) along with EDAC (2.5 equivalent) and DMAP (0.5 equivalent). The reaction mixture was cooled down to −5 °C in an ice bath and continued stirring for 4 h. The progress of each mixture was monitored through thin layered chromatography (TLC), and upon completion, the DCM was evaporated under reduced pressure. The residues were treated with 10% saturated sodium carbonate, and the solid obtained was filtered and washed with water (50 mL) followed by 2N HCl (10 mL) and water (50 mL) to give the desired compounds. The products were recrystallized by aqueous ethanol to obtain in pure form.

((1E,3Z,6E)-3-Hydroxy-5-oxohepta-1,3,6-triene-1,7-diyl)bis(2-methoxy-4,1-phenylene) bis(2,2-dichloroacetate) (**CMC1**) (See Supplementary Material).

Light yellow amorphous, yield: 90%, m.p. 145–147 °C; ^1^H NMR (DMSO-d*_6_*) δ: 7.67 (d, *J* = 15.8 Hz, 2H), 7.59 (bs, 2H), 7.39 (d, *J* = 10.2 Hz, 2H), 7.28 (d, *J* = 8.2 Hz, 2H), 7.23 (s, 2H), 7.04 (d, *J* = 15.8 Hz, 2H), 6.21 (s, 1H), 3.87 (s, 6H). ^13^C NMR (DMSO-d*_6_*) δ: 183.1, 165.8, 162.7, 150.8, 139.8, 139.5, 134.8, 125.2, 122.6, 121.5, 112.5, 101.9, 65.8, 64.5, 56.3. HPLC (CHIRIBIOTIC T 15 cm × 4.6 mm in acetonitrile and water): 95.4%. HRMS *m*/*z* for C_25_H_20_Cl_4_O_8_ [M+H]^+^ Calcd. 588.9945. Found: 588.9984.

### 3.2. General Method for Preparation of CUR–DCA Hybrid Conjugates with an AA Linker (**CMC2–6**)

A dried round-bottom flask containing a small stir bar was charged with CUR (1.0 equivalent) and the respective DCA-amino acid conjugates (2.0 equivalent) dissolved in DCM (5 mL) along with EDAC (2.5 equivalent) and DMAP (0.5 equivalent). The reaction mixture was cooled down to −5 °C in an ice bath and stirred for 4–6 h. The DCA-amino acid conjugates were synthesized following our previously reported method (CBDD). The progress of each mixture was monitored through thin layered chromatography (TLC), and upon completion, the DCM was evaporated under reduced pressure. The residues were treated with 10% saturated sodium carbonate, and the solid obtained was filtered and washed with water (50 mL) followed by 2N HCl (10 mL) and water (50 mL) to give the desired compounds. The products were recrystallized by aqueous ethanol to obtain their pure form.

((1E,3Z,6E)-3-Hydroxy-5-oxohepta-1,3,6-triene-1,7-diyl)bis(2-methoxy-4,1-phenylene) bis(2-(2,2-dichloroacetamido)acetate) (**CMC2**) (See Supplementary Material).

Bright yellow amorphous, yield: 92%, m.p. 167–16975 °C; ^1^H NMR (DMSO-d*_6_*) δ: 9.19 (bs, 2H), 7.65 (d, *J* = 15.8 Hz, 2H), 7.54 (bs, 2H), 7.34 (d, *J* = 8.4 Hz, 2H), 7.17 (d, *J* = 8.2 Hz, 2H), 7.00 (d, *J* = 15.8 Hz, 2H), 6.63 (s, 2H), 6.20 (s, 1H), 4.27 (d, *J* = 5.8 Hz, 4H), 3.85 (s, 6H). ^13^C NMR (DMSO-d*_6_*) δ: 183.2, 167.3, 164.3, 151.0, 139.8, 140.4, 139.7, 134.0, 129.7, 124.8, 123.1, 121.4, 112.3, 101.8, 66.3, 56.1, 41.1. HPLC (CHIRIBIOTIC T 15 cm × 4.6 mm in acetonitrile and water): 96.6%. HRMS *m*/*z* for C_29_H_26_Cl_4_N_2_O_10_ [M+Na]^+^ Calcd. 727.0342. Found: 727.0249.

((1E,3Z,6E)-3-Hydroxy-5-oxohepta-1,3,6-triene-1,7-diyl)bis(2-methoxy-4,1-phenylene) (2S,2’S)-bis(2-(2,2-dichloroacetamido)propanoate) (**CMC3**) (See Supplementary Material).

Yellow amorphous, yield: 89%, m.p. 92–93 °C; ^1^H NMR (DMSO-d*_6_*) δ: 9.24 (d, *J* = 6.6 Hz, 2H), 7.65 (d, *J* = 15.8 Hz, 2H), 7.54 (bs, 2H), 7.35 (d, *J* = 8.2 Hz, 2H), 7.15 (d, *J* = 8.0 Hz, 2H), 7.00 (d, *J* = 15.8 Hz, 2H), 6.52 (s, 2H), 6.21 (s, 1H), 4.63–4.57 (m, 2H), 3.85 (s, 6H), 1.52 (d, *J* = 7.0 Hz, 6H). ^13^C NMR (DMSO-d*_6_*) δ: 183.5, 170.3, 164.0, 151.5, 141.1, 140.2, 134.4, 125.2, 123.5, 121.9, 112.7, 102.2, 66.8, 56.6, 48.9, 17.2. HPLC (CHIRIBIOTIC T 15 cm × 4.6 mm in acetonitrile and water): 98.6%. HRMS *m*/*z* for C_31_H_30_Cl_4_N_2_O_10_ [M+H]^+^ Calcd. 731.0655. Found: 731.0733.

((1E,3Z,6E)-3-Hydroxy-5-oxohepta-1,3,6-triene-1,7-diyl)bis(2-methoxy-4,1-phenylene) bis(3-(2,2-dichloroacetamido)propanoate) (**CMC4**) (See Supplementary Material).

Light yellow amorphous, yield: 93%, m.p. 140–142 °C; ^1^H NMR (DMSO-d*_6_*) δ: 8.79 (bs, 2H), 7.66 (d, *J* = 15.8 Hz, 2H), 7.52 (bs, 2H), 7.34 (bs, 2H), 7.18 (d, *J* = 6.3 Hz, 2H), 7.00 (d, *J* = 15.8 Hz, 2H), 6.51 (s, 2H), 6.20 (s, 1H), 3.85 (s, 6H), 3.49 (s, 4H), 2.82 (s, 4H). ^13^C NMR (DMSO-d*_6_*) δ: 183.7, 169.6, 164.3, 151.6, 141.2, 140.3, 134.3, 125.1, 123.8, 121.8, 112.5, 102.2, 67.2, 56.5, 36.0, 33.4. HPLC (CHIRIBIOTIC T 15 cm × 4.6 mm in acetonitrile and water): 95.6%. HRMS *m*/*z* for C_31_H_30_Cl_4_N_2_O_10_ [M+H]^+^ Calcd. 731.0655. Found: 731.0545.

((1E,3Z,6E)-3-Hydroxy-5-oxohepta-1,3,6-triene-1,7-diyl)bis(2-methoxy-4,1-phenylene) (2S,2’S)-bis(2-(2,2-dichloroacetamido)-3-phenylpropanoate) (**CMC5**) (See Supplementary Material).

Yellow amorphous, yield: 90%, m.p. 192–194–75 °C; ^1^H NMR (DMSO-d*_6_*) δ: 9.27 (d, *J* = 7.2 Hz, 2H), 7.66 (d, *J* = 15.8 Hz, 2H), 7.56 (bs, 2H), 7.35–7.25 (m, 11H), 7.12 (d, *J* = 7.8 Hz, 2H), 7.02 (d, *J* = 15.8 Hz, 2H), 6.50 (s, 2H), 6.21 (s, 1H), 4.63–4.57 (m, 2H), 3.85 (s, 6H), 1.52 (d, *J* = 7.0 Hz, 6H). ^13^C NMR (DMSO-d*_6_*) δ: 183.6, 169.1, 164.1, 151.5, 140.9, 140.2, 136.8, 134.5, 129.8, 128.8, 127.3, 125.3, 123.5, 121.9, 112.8, 102.3, 66.7, 56.6, 54.4, 36.6. HPLC (CHIRIBIOTIC T 15 cm × 4.6 mm in acetonitrile and water): 97.2%. HRMS *m*/*z* for C_43_H_38_Cl_4_N_2_O_10_ [M+H]^+^ Calcd. 883.1281. Found: 883.1318.

((1E,3Z,6E)-3-Hydroxy-5-oxohepta-1,3,6-triene-1,7-diyl)bis(2-methoxy-4,1-phenylene) bis(4-(2,2-dichloroacetamido)butanoate) (**CMC6**) (See Supplementary Material).

Bright yellow amorphous, yield: 96%, m.p. 130–132 °C; ^1^H NMR (DMSO-d*_6_*) δ: 8.68 (bs, 2H), 7.65 (d, *J* = 15.8 Hz, 2H), 7.52 (bs, 2H), 7.34 (d, *J* = 8.7 Hz, 2H), 7.17 (d, *J* = 8.2 Hz, 2H), 6.99 (d, *J* = 15.8 Hz, 2H), 6.46 (s, 2H), 6.20 (s, 1H), 3.84 (s, 6H), 3.27–3.30 (m, 4H), 2.61 (t, *J* = 7.5 Hz, 4H), 1.83 (t, *J* = 7.2 Hz, 4H). ^13^C NMR (DMSO-d*_6_*) δ: 183.2, 170.6, 163.7, 151.1, 140.9, 139.8, 133.7, 124.6, 123.3, 121.4, 112.1, 101.7, 66.9, 56.0, 38.5, 30.5, 24.0. HPLC (CHIRIBIOTIC T 15 cm × 4.6 mm in acetonitrile and water): 98.6%. HRMS *m*/*z* for C_33_H_34_Cl_4_N_2_O_10_ [M+Na]^+^ Calcd. 783.0968. Found: 783.0809.

### 3.3. MTT Assay

MCF10A, T47D, and MB231 cells (5 × 10^3^) were seeded in 96-well plates and incubator at 37 °C with 5% CO_2_ in MEGM, DMEM, and RPMI medium (100 μL), respectively. After 24 h, the medium was replaced with the CMC conjugates at different concentrations (0, 1, 5, and 10 μM) for 72 h. After 72 h, 10 μL MTT reagent was added to each well and incubated for 2 h for the formation of purple formazan and then added 100 μL detergent to dissociate the formazan precipitate and measured at 570 nm. Values are shown as mean ± SD of three experiments with 3 wells in each, a total of 9 repeats [28].

### 3.4. Colony Formation Assay 

MCF10A, T47D, and MB231 cells (5 × 10^3^) were seeded in 24-well plates, and cells were exposed to different CMC conjugates at different concentrations (0, 1, 5, and 10 μM) for 2 weeks, changing the medium for every 3 days with respective CMC conjugates at the indicated concentrations. After 2 weeks, cells were washed with PBS and fixed in 100% methanol for 30 min followed by staining with KaryoMax Giemsa stain for 1 h. The unfound dyes were removed by washing the wells with water and dried overnight at room temperature. Finally, cells were lysed with lysis buffer (1% SDS in 0.2 N NaOH) for 5 min, and the absorbance of the released dye was measured at 630 nm, as described before [28]. Values are shown as mean ± SD of three experiments with 3 wells in each, for a total of 9 repeats.

### 3.5. Institutional Compliance 

The animal experiments reported in this study were approved by the Augusta University IACUC (protocol #2015-0737, approval date 30 July 2021) and Biosafety (protocol #1462, approval date 30 July 2021) Committees.

### 3.6. Cell Lines 

The human non-transformed normal mammary epithelial cell line MCF10A was obtained from the American Type Culture Collection (ATCC). The estrogen receptor-positive breast cancer (ER + BC) cell line MCF7 and triple-negative breast cancer (TNBC) cell line MDA-MB231 (MB231) were obtained from ATCC. Cell lines from ATCC have been thoroughly tested and authenticated, and morphology, karyotyping, and PCR-based approaches were used to confirm the identity of the cell lines. The MCF10A cells were grown in MEGM complete medium; MCF7 cells were grown in DMEM medium with 10% FBS; MDA-MB-231 cells were grown in Leibovitz’s L-15 medium with 10% FBS and 1% P/S. All these cell lines have been routinely tested for mycoplasma contamination using the Universal mycoplasma detection kit obtained from ATCC (Manassas, VA, USA), and the last mycoplasma test was performed in July 2021. Mycoplasma-free cell lines were used in all our experiments.

### 3.7. Animals 

C57BL/6 (Stock #000664) and MMTV–PyMT–Tg (Stock #002374) mice were obtained from the Jackson laboratories. All these mice were bred and maintained in Augusta University Animal Facility by the guidelines of the Institutional Animal Care Use Committees. All euthanasia protocols were performed by the regulations and guidelines presented by IACUC and LAS of Augusta University.

Administration of CMC compounds to the mice: For toxicity studies, CMC1 and CMC2 conjugates at two different concentrations (50 and 100 mg/kg body) were given oral gavage daily for 7 days. At the end of the experiment, animals were euthanized, kidneys, liver, and lungs were analyzed for morphological changes. We also collected blood samples to measure Creatinine using a Creatinine assay kit (obtained from Millipore Sigma, Burlington, MA, USA, Catalog # MAK080), Alanine aminotransferase (ALT), using an ALT assay kit (obtained from the Millipore Sigma, Catalog #MAK053), Aspartate aminotransferase (AST), using an AST assay kit obtained from Millipore Sigma, Catalog #MAK055), and blood urea and nitrogen (BUN), using a urea nitrogen assay kit (obtained from ThermoFisher Scientific, Waltham, MA, USA, Catalog #EIABUN) as per the manufacturer’s instruction.

Similarly, six-week-old MMTV–PyMT–Tg mice were grouped into two groups—one control and one CMC2 treatment. The control mice received PBS, and the CMC2-treated group received CMC2 conjugate (10 mg/kg body by oral gavage, three times a week) for 7 weeks. We monitored animal weight and measured the tumor volume twice a week. At the end of the experimental period, mice were euthanized, and tumor tissues were harvested and measured for tumor weight. Tumor tissues were processed to extract RNA and protein and fixed in 10% buffered formalin phosphate solution (obtained from Fisher Scientific, Catalog #SF100-4) for morphological analysis.

### 3.8. Statistical Analysis

Statistical analysis was done using one-way ANOVA followed by the Bonferroni multiple comparison test and also using Student’s *t*-test with the two-tail distribution. The software used was Graph Pad Prism, version 8.0, San Diego, CA, USA. A value of *p* < 0.05 was considered statistically significant. GraphPad, Sigma Plot, and Excel programs were used to draw figures.

## 4. Conclusions

CUR–DCA hybrid conjugates **CMC1-6** were synthesized in good yields by an optimized facile reaction condition. Two of the synthesized conjugates (**CMC1** and **CMC2**) exhibit enhanced anticancer properties against BC with reduced possible toxicity to human normal immortalized mammary epithelial cell line (MCF10A) and normal C57BL/6 mice. Animal studies suggest **CMC2** is a highly effective and safer therapeutic agent for BC. We believe the hybrid conjugates work as an effective prodrug of curcumin, and DCA provides a synergistic effect. The molecular docking and ADME studies support the drug candidacy of **CMC2** for BC. The potential conjugates need further investigation with different animal models to develop the pharmacokinetic profile to better understand the molecular mechanism and develop safer as well as efficient oral drug candidates for BC.

## Figures and Tables

**Table 1:** EC_50_ values for CMC compounds were determined by a clonogenic survival assay for two human BC cell lines.

Name of the Compound	EC50 for MCF10A	EC_50_ for T47D Cells	EC_50_ for MB231 Cells
**CMC1**	8.982 μM	1.648 μM	0.4240 μM
**CMC2**	9.675 μM	1.421 μM	0.7780 μM
**CMC3**	9.714 μM	1.595 μM	0.5179 μM
**CMC4**	8.859 μM	1.255 μM	1.1320 μM
**CMC5**	9.604 μM	1.245 μM	0.8375 μM
**CMC6**	9.474 μM	1.372 μM	0.9418 μM

**Table 2:** Molecular docking and predicted ADME properties of CMC compounds.

Name	CMC Docking with DYRK2		dG kJ/mol	Score	L.E
CUR	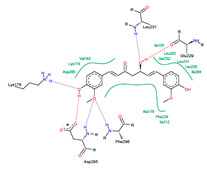	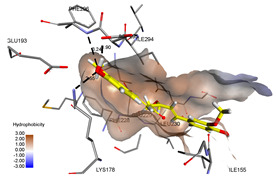	−24	−29.24	0.22
**CMC1**	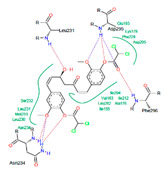	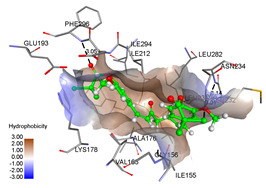	−27	−22.86	0.18
**CMC2**	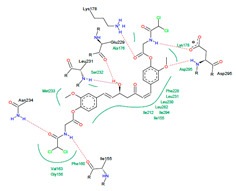	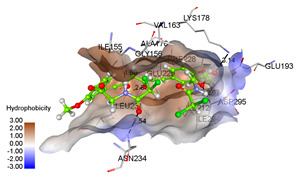	−67	−33.02	0.35
**CMC3**	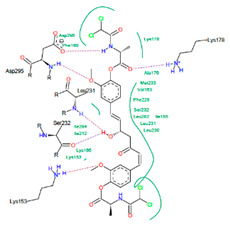	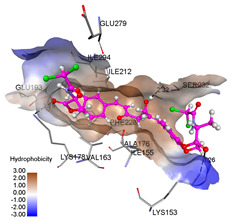	−21	−16.71	0.11
**CMC4**	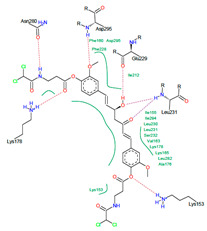	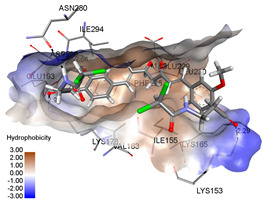	−49	−25.09	0.25
**CMC6**	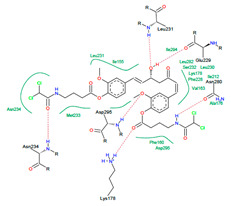	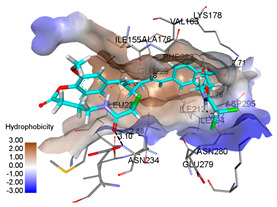	−58	−21.10	0.28

**Table 3:** Predicted ADME properties of CMC compounds.

Name	Log P	Aq. Sol (log mol/L)	HERG II Inhibitor	Dev Tox	CYP2D6 Substrate	P-gp Substrate	HIA %
CUR	3.852	−3.878	+	+	Med	-	84.38
**CMC1**	5.859	−4.644	-	-	Low	-	81.65
**CMC2**	4.092	−4.031	-	-	Low	+	66.25
**CMC3**	4.869	−4.010	-	-	Low	+	68.18
**CMC4**	4.872	−3.700	-	+	Low	+	61.82
**CMC5**	7.314	−2.981	+	+	Med	-	81.50
**CMC6**	5.65	−3.336	-	+	Med	+	67.89

## Data Availability

Data is contained within the article and Supplementary Material.

## Supplementary Materials

The following are available online at https://www.mdpi.com/article/10.3390/ph15040451/s1, ^1^H NMR and ^13^C NMR of all the synthesized compounds.
