# Supplementary material for: Design, Synthesis, and Molecular Docking Studies of Curcumin Hybrid Conjugates as Potential Therapeutics for Breast Cancer
Source: Pharmaceuticals (Basel). 2022 Apr 6;15(4):451. doi: 10.3390/ph15040451 (PMC9028889; doi:10.3390/ph15040451)
Supplement: Supplementary file 1 [file pharmaceuticals-15-00451-s001.zip › pharmaceuticals-1662348-supplementary.pdf]

Supplementary Material

Design, Synthesis, and Molecular Docking Studies of Curcumin Hybrid Conjugates as Potential Therapeutics for Breast Cancer

Siva S. Panda [1,*], Queen L. Tran [1], Pragya Rajpurohit [2,3], Girinath G. Pillai [4], Sean J. Thomas [1], Allison E. Bridges [2,3], Jason E. Capito [1], Muthusamy Thangaraju [2,3,*] and Bal L. Lokeshwar [2,3,5]

[1] Department of Chemistry and Physics, Augusta University, Augusta, GA 30912, USA; queentran29@gmail.com (Q.L.T.); seanjosephthomas@gmail.com (S.J.T.); jcaps79@gmail.com (J.E.C.)

[2] Department of Biochemistry and Molecular Biology, Augusta University, Augusta, GA 30912, USA; pragyaraj2018@gmail.com (P.R.); allison.bridges@ngu.edu (A.E.B.); blokeshwar@augusta.edu (B.L.L.)

[3] Georgia Cancer Center, Augusta, GA 30912, USA

[4] Discovery Chemistry Nyro Research India, Kochi 682021, India; giribio@gmail.com

[5] Department of Medicine, Medical College of Georgia, Augusta University, Augusta, GA 30912, USA

* Correspondence: sipanda@augusta.edu (S.S.P.); mthangaraju@augusta.edu (M.T.)

1H NMR and 13C NMR of all the synthesized compounds.

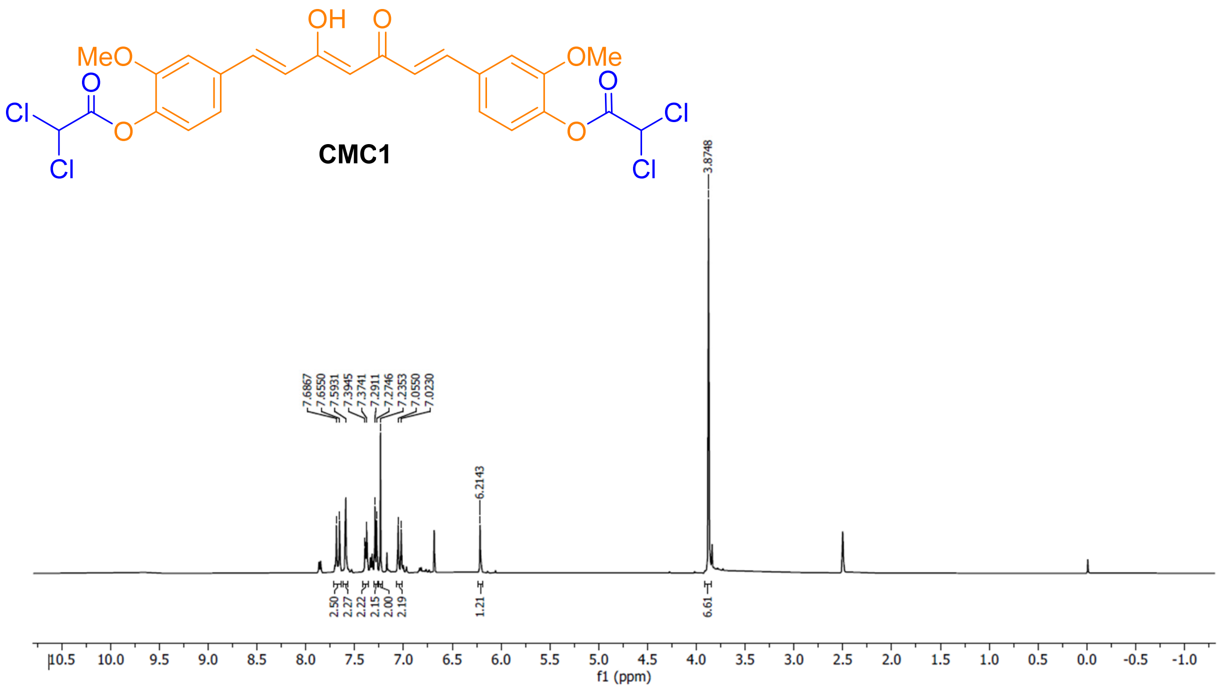

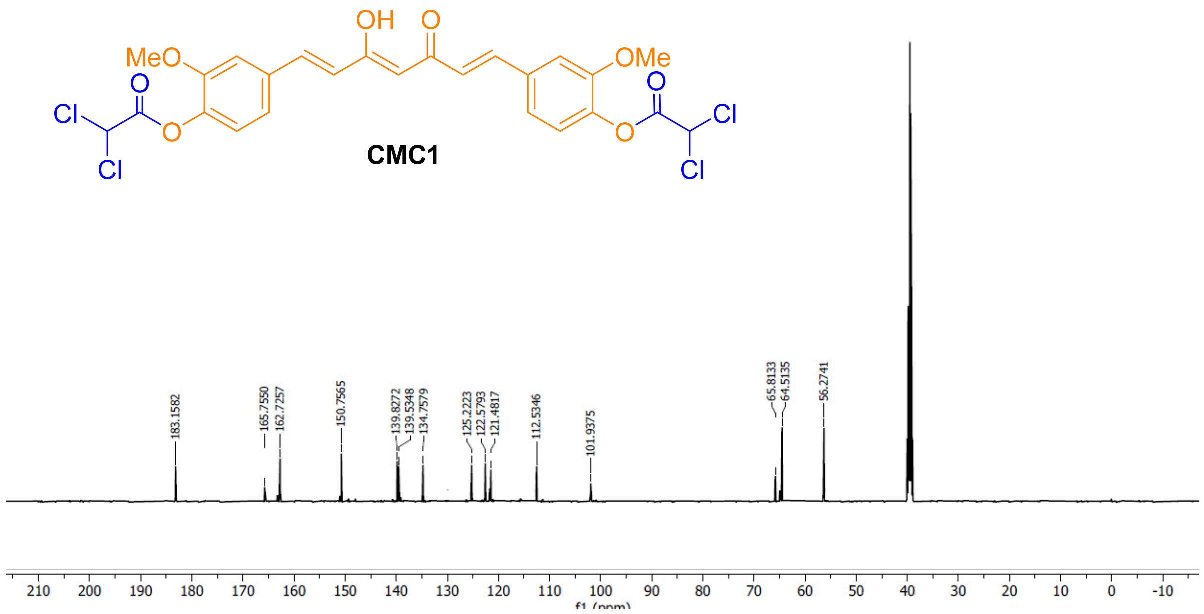

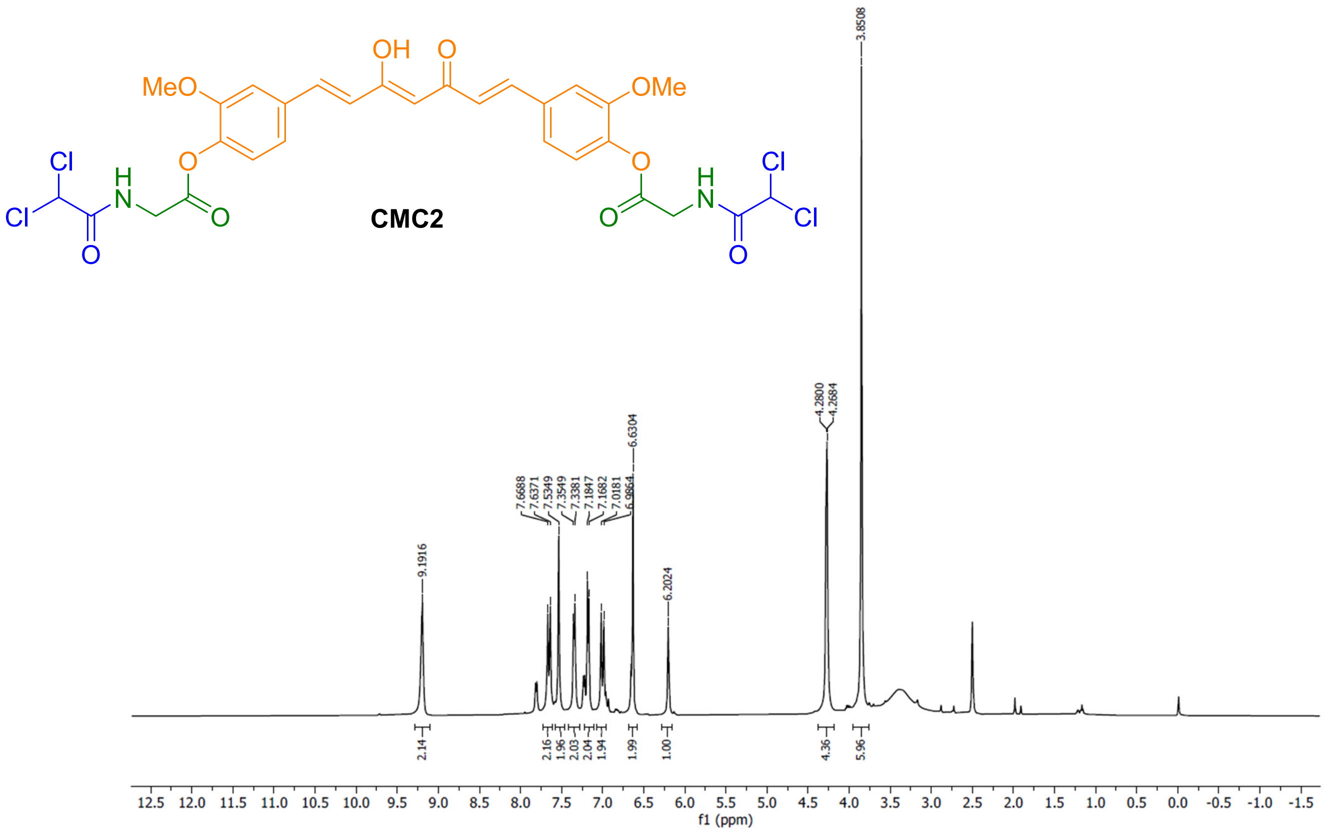
MeO
OH
O
OMe
Cl
H
N
O
O
CMC2
3.8508
4.2800
4.2684
6.6304
7.6688
7.6371
7.5349
7.3549
7.3381
7.1847
7.1682
7.0181
6.9864
9.1916
6.2024
2.14
2.16
1.96
2.03
2.04
1.94
1.99
1.00
4.36
5.96
12.5 12.0 11.5 11.0 10.5 10.0 9.5 9.0 8.5 8.0 7.5 7.0 6.5 6.0 5.5 5.0 4.5 4.0 3.5 3.0 2.5 2.0 1.5 1.0 0.5 0.0 -0.5 -1.0 -1.5
f1 (ppm)

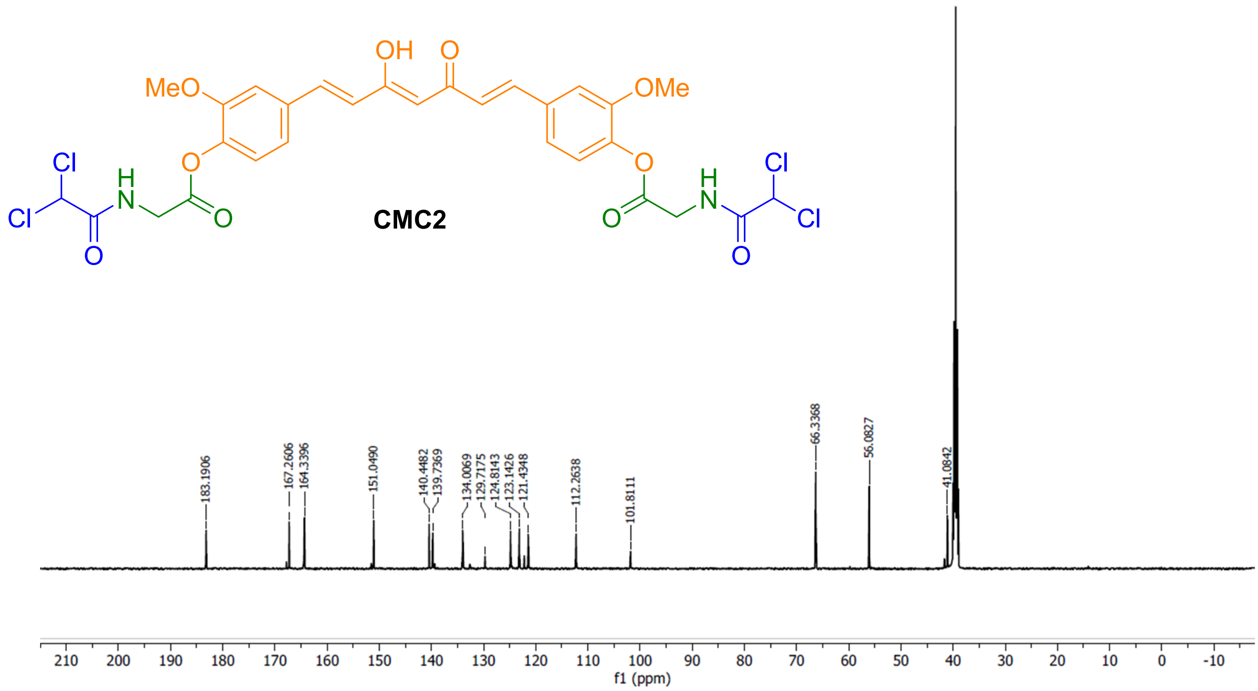
MeO
OH
O
OMe
Cl
H
N
O
O
CMC2
183.1906
167.2606
164.3396
151.0490
140.4482
139.7369
134.0069
129.7175
124.8143
123.1426
121.4348
112.2638
101.8111
66.3368
56.0827
41.0842
210 200 190 180 170 160 150 140 130 120 110 100 90 80 70 60 50 40 30 20 10 0 -10
f1 (ppm)

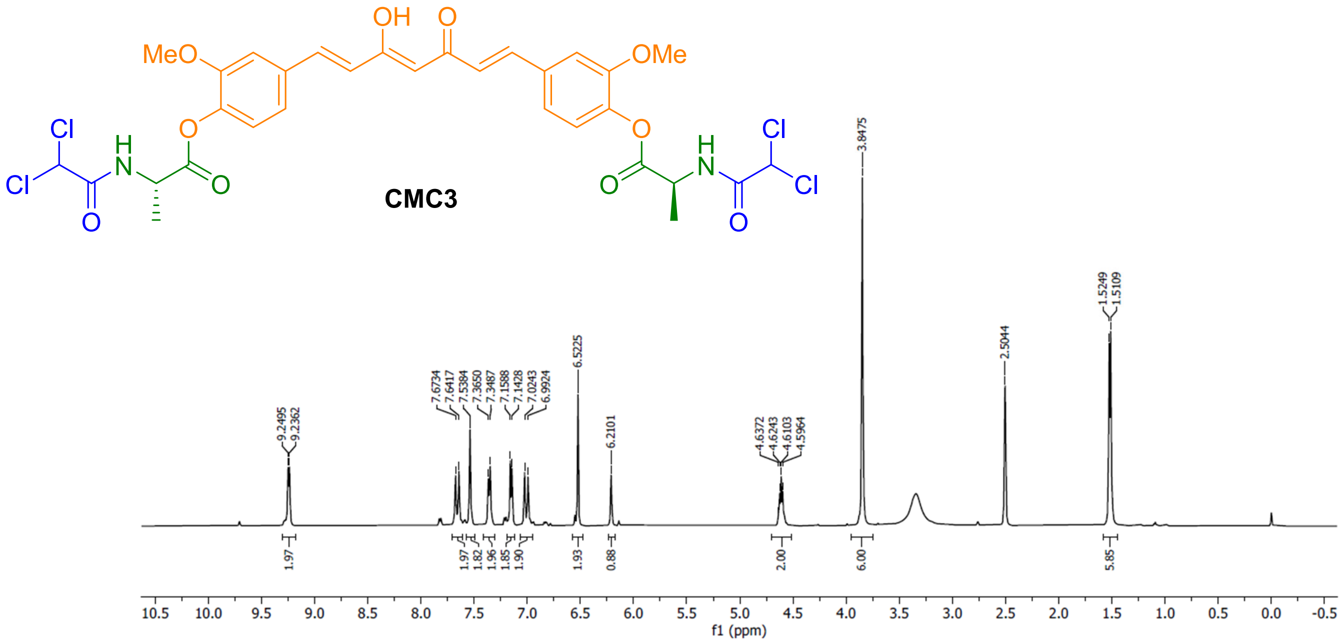
CMC3
MeO
OMe
OH
O
Cl
H
N
f1 (ppm)

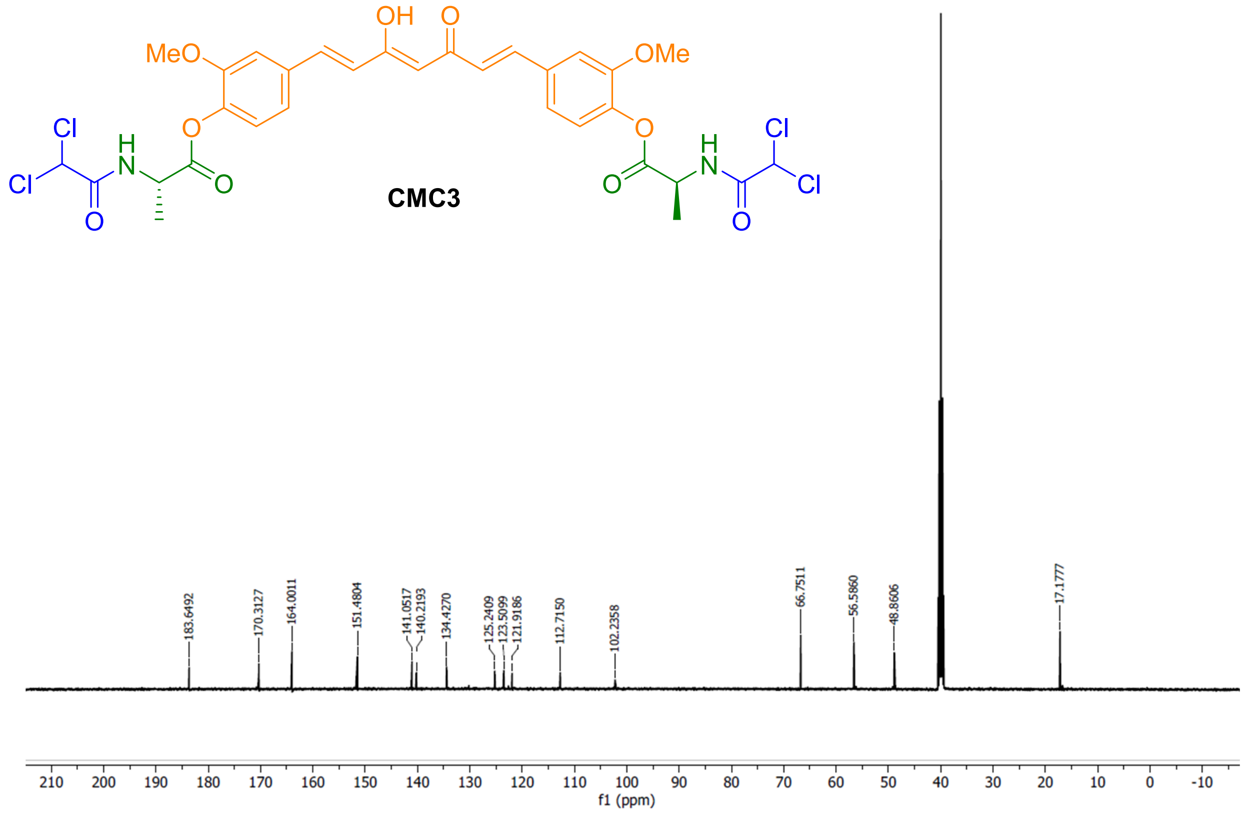
CMC3
MeO
OMe
OH
O
Cl
H
N
f1 (ppm)

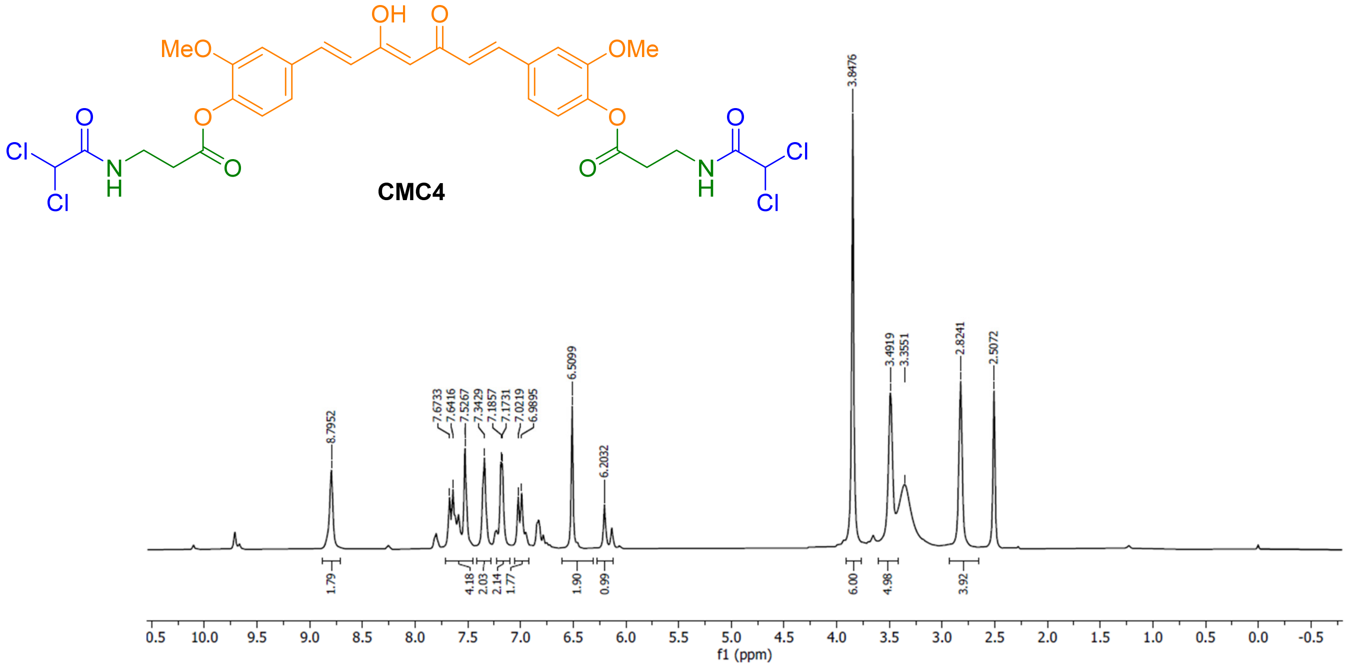
OH
O
MeO
OMe
O
Cl
N
H
Cl
CMC4
8.7952
7.6733
7.6416
7.5267
7.3429
7.1857
7.1731
7.0219
6.9895
6.5099
6.2032
3.8476
3.4919
3.3551
2.8241
2.5072
1.79
4.18
2.03
2.14
1.77
1.90
0.99
6.00
4.98
3.92
f1 (ppm)

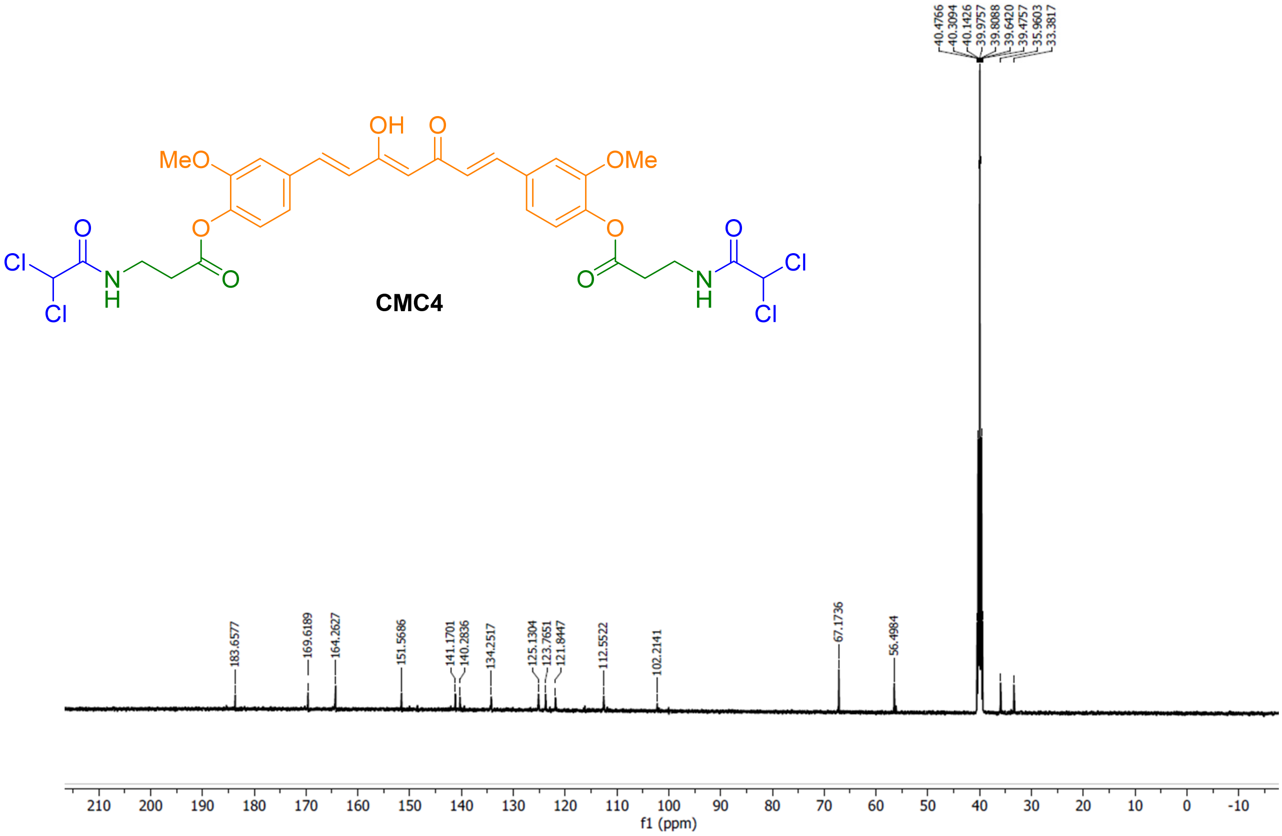
OH
O
MeO
OMe
O
Cl
N
H
Cl
CMC4
40.4766
40.3094
40.1426
39.9757
39.8088
39.6420
39.4757
35.9603
33.3817
183.6577
169.6189
164.2627
151.5686
141.1701
140.2836
134.2517
125.1304
123.7651
121.8447
112.5522
102.2141
67.1736
56.4984
f1 (ppm)

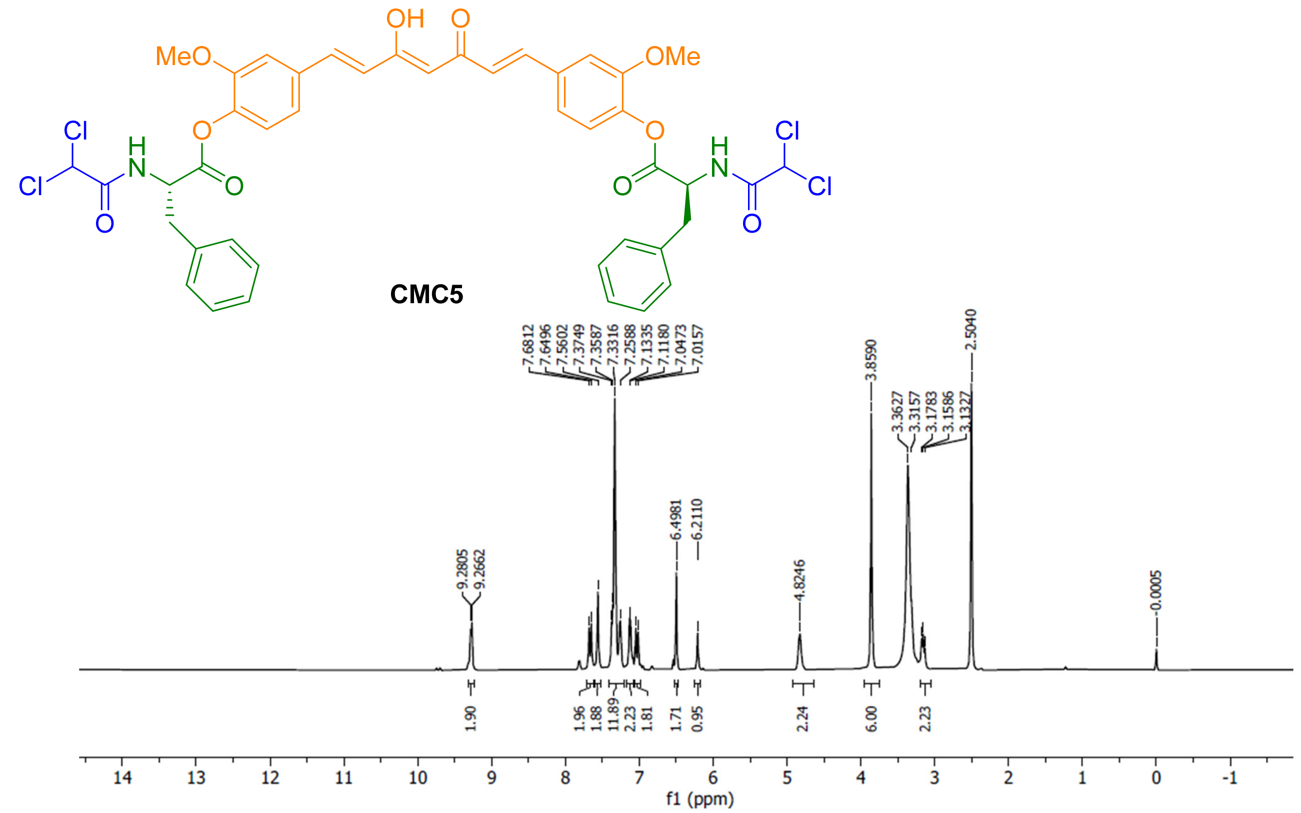
OH
O
MeO
OMe
O
H
N
Cl
Cl
O
O
CMC5
9.2805
9.2662
7.6812
7.6496
7.5602
7.3749
7.3587
7.3316
7.2588
7.1335
7.1180
7.0473
7.0157
6.4981
6.2110
4.8246
3.8590
3.3627
3.3157
3.1783
3.1586
3.1327
2.5040
-0.0005
1.90
1.96
1.88
11.89
2.23
1.81
1.71
0.95
2.24
6.00
2.23
f1 (ppm)

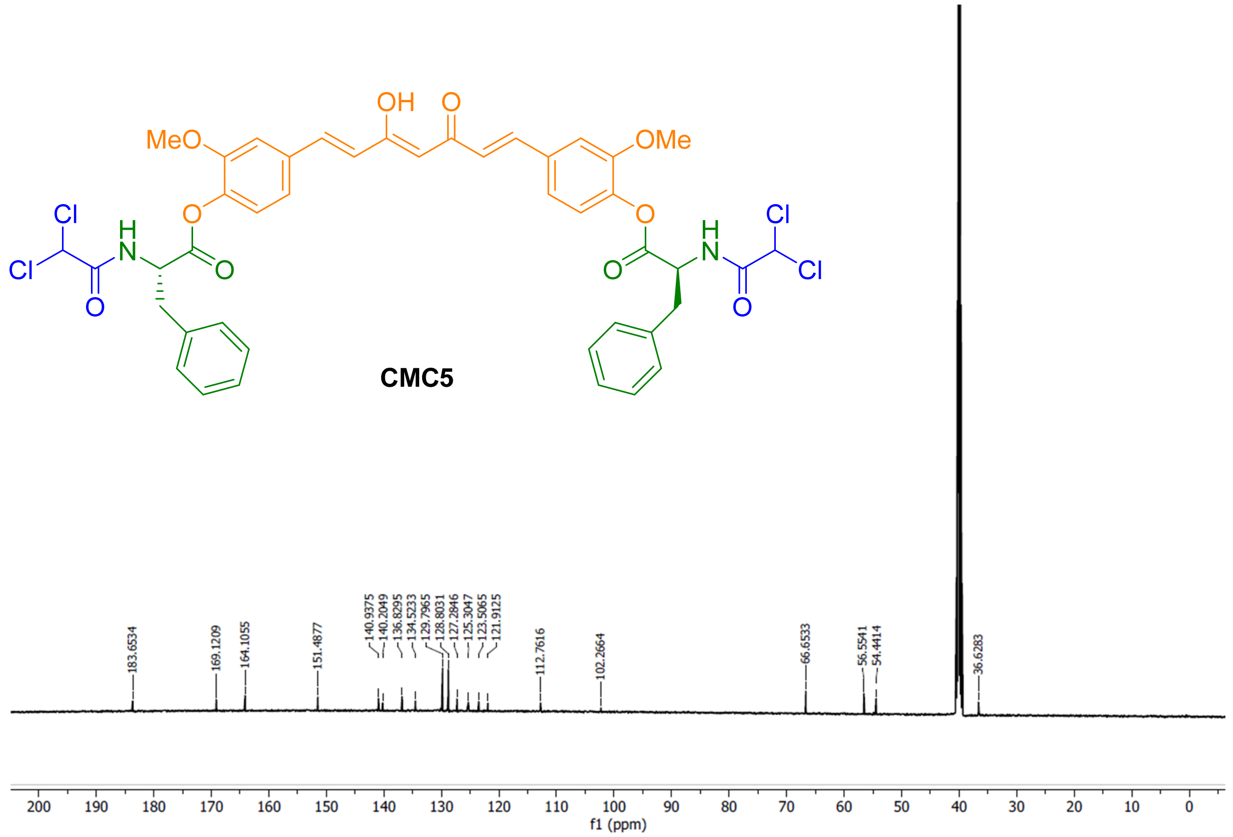
OH
O
MeO
OMe
O
H
N
Cl
Cl
O
O
CMC5
183.6534
169.1209
164.1055
151.4877
140.9375
140.2049
136.8295
134.5233
129.7965
128.8031
127.2846
125.3047
123.5065
121.9125
112.7616
102.2664
66.6533
56.5541
54.4414
36.6283
f1 (ppm)

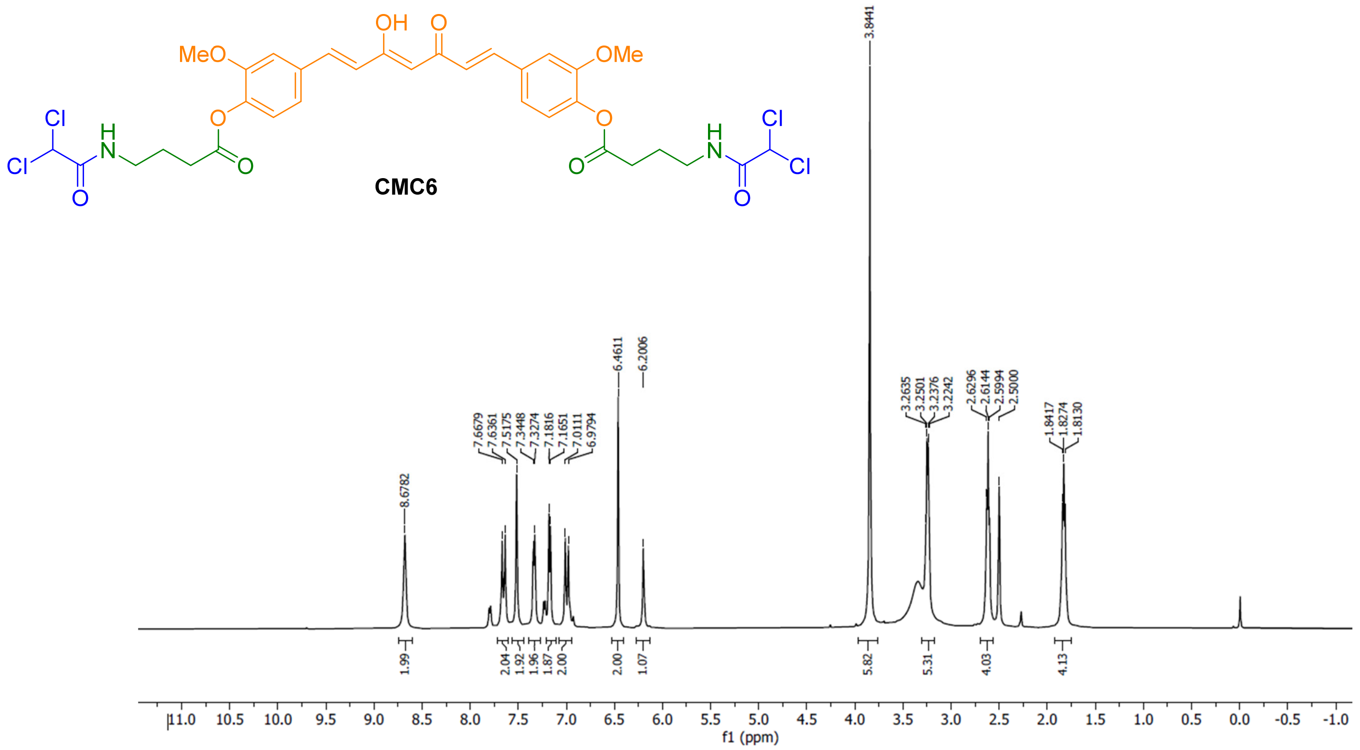

CMC6
f1 (ppm)

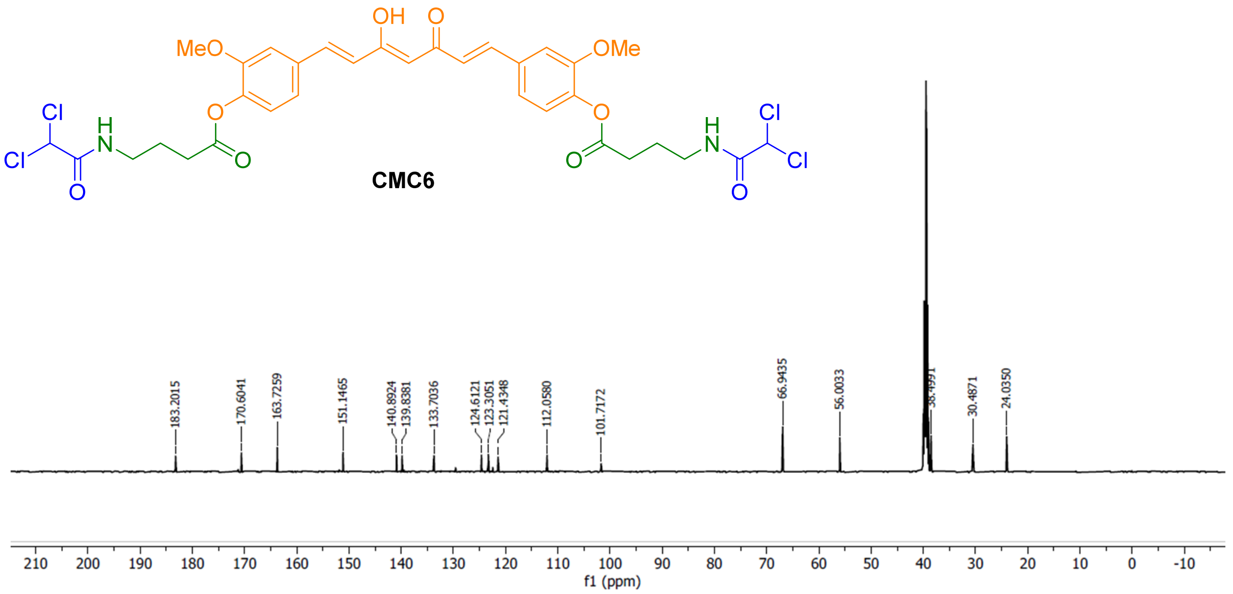

CMC6
f1 (ppm)
